# Effective Construction of High-quality Iron Oxy-hydroxides and Co-doped Iron Oxy-hydroxides Nanostructures: Towards the Promising Oxygen Evolution Reaction Application

**DOI:** 10.1038/srep43590

**Published:** 2017-03-08

**Authors:** Xinyu Zhang, Li An, Jie Yin, Pinxian Xi, Zhiping Zheng, Yaping Du

**Affiliations:** 1 Frontier Institute of Science and Technology jointly with College of Science, State Key Laboratory for Mechanical Behavior of Materials, Xi’an Jiaotong University, Xi’an 710049, P. R. China; 2 Key Laboratory of Nonferrous Metal Chemistry and Resources Utilization of Gansu Province, State Key Laboratory of Applied Organic Chemistry and College of Chemistry and Chemical Engineering, Research Center of Biomedical Nanotechnology, Lanzhou University, Lanzhou, 730000, P. R. China; 3 Department of Chemistry and Biochemistry, The University of Arizona, Tucson, AZ 85721-0041, USA

## Abstract

Rational design of high efficient and low cost electrocatalysts for oxygen evolution reaction (OER) plays an important role in water splitting. Herein, a general gelatin-assisted wet chemistry method is employed to fabricate well-defined iron oxy-hydroxides and transitional metal doped iron oxy-hydroxides nanomaterials, which show good catalytic performances for OER. Specifically, the Co-doped iron oxy-hydroxides (Co_0.54_Fe_0.46_OOH) show the excellent electrocatalytic performance for OER with an onset potential of 1.52 V, tafel slope of 47 mV/dec and outstanding stability. The ultrahigh oxygen evolution activity and strong durability, with superior performance in comparison to the pure iron oxy-hydroxide (FeOOH) catalysts, originate from the branch structure of Co_0.54_Fe_0.46_OOH on its surface so as to provide many active edge sites, enhanced mass/charge transport capability, easy release oxygen gas bubbles, and strong structural stability, which are advantageous for OER. Meanwhile, Co-doping in FeOOH nanostructures constitutes a desirable four-electron pathway for reversible oxygen evolution and reduction, which is potentially useful for rechargeable metal−air batteries, regenerative fuel cells, and other important clean energy devices. This work may provide a new insight into constructing the promising water oxidation catalysts for practical clean energy application.

Nowadays, the urgent demands of clean energy have been stirred up in the exploration of sustainable energy production with high efficiency, low cost, and environmental benignity^1^,^2^,^3^,^4^. Water splitting, which could develop energy storage and conversion devices, has been recognized as one of the key technological candidates to meet the ever-growing sustainable energy demands. However, the efficiency of economical water splitting is under restriction mostly due to the high overpotential interrelated to the occurrence of oxygen evolution reaction (OER). Despite of recent advances in the utilization of various nanostructured catalysts, such as commonly used noble metal Ru and Ir-based nanocatalysts, the exploration of novel catalysts with low cost and high activities to enhance the OER efficiency still remains a big challenge.

In the past few decades, many efforts have been made to solve this challenge by using transitional metals with low cost such as Fe, Co and Ni-based catalysts to substitute noble metal nanocatalysts to expedite OER efficiency^5^,^6^. Recently, as one of the most important transitional-metal-based nanocatalysts ion oxy-hydroxides (FeOOH) with open structure, low cost, natural abundance, and environmental friendliness of iron^7^ have been gradually acknowledged and further explored for OER application^8^,^9^. However, the poor electrical conductivity of the FeOOH (~10^−5^ S cm^−1^) remains a major challenge and limits its mass-transfer kinetics^8^. Thus, recently, some studies have tried to address this issue by forming hybrid FeOOH nanomaterials^10^,^11^,^12^. Among them, Co doping in FeOOH nanostructure had shown excellent OER performance, because the Co ions could improve electron transfer thus enhance the electrical conductivity^13^. However, in the above cases, fabricate of high-quality FeOOH and Co-doped FeOOH nanostructures with pure phase, monodisperse and well-defined morphology, have not been demonstrated, which stimulate the continuous and systematic exploration.

Gelatin as a water-soluble collagen, consisting of N-H functional groups, possesses many advantages to form inorganic-organic template for manipulating the growth of inorganic nanomaterials with diverse novel structures^14^,^15^,^16^. Particularly, the molecule of gelatin is composed of periodic repetitions of amino acid sequences, *i.e.* glycine-proline-hydroxyproline segments, where the constituent N-H functional groups trend to interact strongly with metal ions *via* multiple nitrogen coordination reactions^17^,^18^,^19^,^20^,^21^.

Owing to gelatin’s unique structural features and tunable properties, in the present work, we chose gelatin as the soft-template to synthesize high-quality FeOOH and Co-doped FeOOH nanostructures (Co_x_Fe_1−x_OOH (*x* = 0.23, 0.54, 0.77)) (Fig. 1). The Co_0.54_Fe_0.46_OOH hybrid had lower onset potential of 1.52 V, lower overpotential of 390 mV at current density of 10 mA/cm^2^, smaller tafel slope of 47 mV/dec and fairly longer time stability of 25000 s than other contrast catalysts, which can be attributed to more active edge sites, enhanced mass/charge transport capability and strong structural stability of the Co_0.54_Fe_0.46_OOH hybrid.

## Results

### Morphologies of FeOOH nanostructures

The morphologies of FeOOH nanostructures were characterized by transmission electron microscopy (TEM) and high-angle annular dark field-scanning transmission electron microscopy (HAADF-STEM). Based on the TEM and HAADF-STEM results, three different morphologies of FeOOH nanostructures with high morphological yields were harvested^22^. Figure 2(a) and (b) show the TEM and HAADF-STEM images of urchin-like FeOOH (termed as FeOOH1) nanostructure. Numerous nanorods, with the average size of ~500 ± 100 nm in length and ~50 ± 10 nm in width, were integrated together to form such three dimensional (3D) hierarchical structures. A digital photo (inset of Fig. 2(b) demonstrates that the FeOOH1 nanostructures were readily dispersed and highly stable in absolute ethanol. Similarly, Fig. 2(c) and (d) exhibit the bowknot-like FeOOH (termed as FeOOH2) nanostructure with the average size of ~700 nm ± 100 nm in length and ~100 nm ± 50 nm in width at the knot position. Figure 2(e) and (f) depict the bamboo leaf-like FeOOH (termed as FeOOH3) nanostructure with the average size of ~500 nm ± 100 nm in length and ~50 nm ± 10 nm in width. Meanwhile, the insets of Fig. 2d and f show the digital photos of FeOOH2 and FeOOH3 nanostructures dispersed in absolute ethanol for more than 1 week, also demonstrating the high stability and dispersibility of FeOOH nanostructures.

### XRD analysis of FeOOH nanostructures

The powder X-ray diffraction (XRD) patterns of FeOOH nanostructures are shown in Fig. 3(a). As indicated in Fig. 3(a), the XRD patterns of FeOOH nanostructures (FeOOH1, FeOOH2 and FeOOH3) were all indexed to a tetragonal phase of Akaganeite (JCPDS: 34–1266, space group: I4/m) with lattice constants: *a* = *b* = 10.54 Å, *c* = 3.03 Å, V = 336.29 Å^3^. Additionally, the broadening of the diffraction peaks suggested the samples were nanocrystalline nature. To study the chemical state of FeOOH nanostructures, the X-ray photoelectron spectroscopy (XPS) analysis is shown in Fig. 3(b) and Fig. S1 in the Supplementary Information. Figure 3(b) illustrates the Fe 2p XPS signals of FeOOH nanostructures, and the peaks located at 711.00 eV and 724.70 eV were ascribed to the core levels of Fe 2p_3/2_ and Fe 2p_1/2_ of FeOOH, respectively. Shakeup satellites located at 718.90 and 733.40 indicating the presence of trivalent Fe in FeOOH samples^22^. The above results suggested that the oxidation states of iron ions are mainly trivalent for the FeOOH nanostructures, which is also in accord with the result of XRD characterization.

### Structure and composition of Co_0.54_Fe_0.46_OOH nanostructures

**Figure 1:**
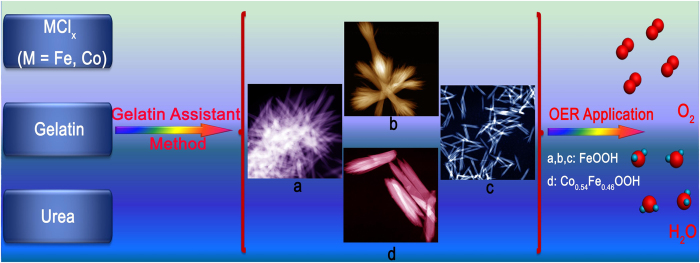
Schematic illustration of the formation of FeOOH and Co-doped FeOOH nanostructures and their OER application.

**Figure 2:**
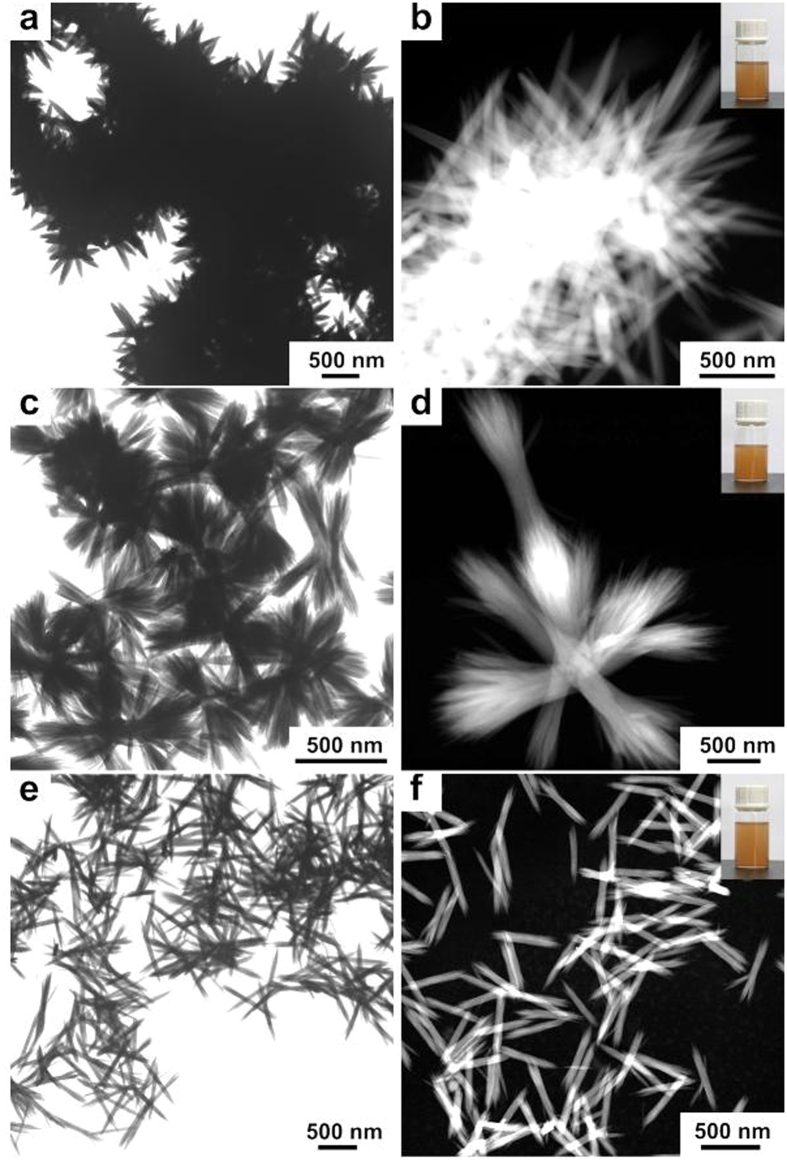
TEM and HAADF-STEM images of (**a**,**b**) FeOOH1, (**c**,**d**) FeOOH2, and (**e**,**f**) FeOOH3 nanostructures. Insets of (**b**),(**d**), and (**f**) are the photos of colloidal FeOOH samples which were dispersed in absolute ethanol and had been placed in an ambient environment for more than 1 week.

**Figure 3:**
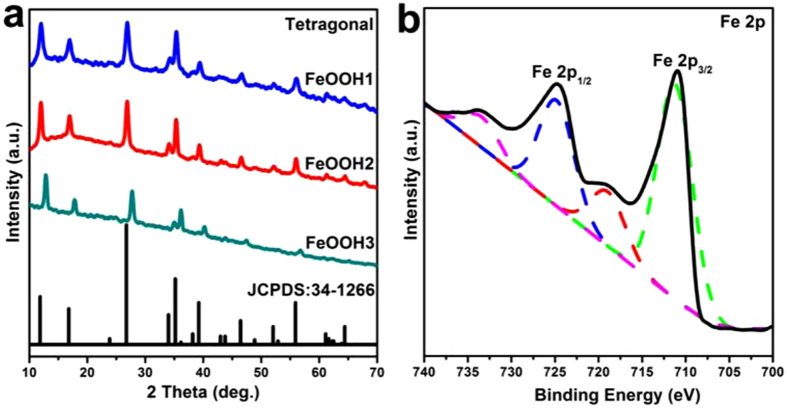
(**a**) XRD patterns of as-prepared FeOOH nanostructures. (**b**) XPS patterns of as-prepared FeOOH nanostructures (Fe 2p).

Specifically, metal oxy-hydroxide compound is an important semiconducting material with unique electrochemical properties^23^,^24^,^25^. As a proof of conception application, our FeOOH samples with diverse nanostructures were used for OER catalyst application (Fig. 1). Meanwhile, recent studies showed that the doping of transitional metals, such as Co doped into oxy-hydroxide based OER catalysts can improve electron transfer, reduce tafel slope, and increase electric conductivity, thus enhancing the OER performances^26^. Herein, we employed the gelatin-assisted wet chemistry method to obtain Co doped FeOOH nanocatalysts (Co_x_Fe_1−x_OOH) and investigated their OER performances. Based on our study, the Co_0.54_Fe_0.46_OOH (*x* = 0.54) had the highest OER catalytic activity.

**Figure 4:**
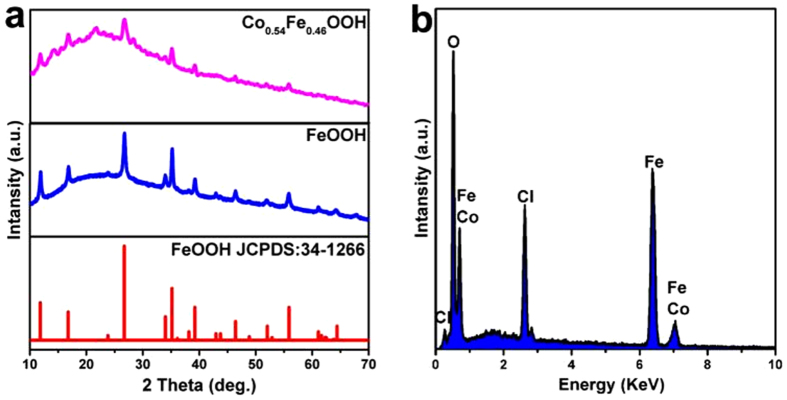
(**a**) XRD patterns of as-prepared Co_0.54_Fe_0.46_OOH. (**b**) The energy-dispersive x-ray spectrum (EDS) spectra of Co_0.54_Fe_0.46_OOH.

The XRD patterns of Co doped FeOOH nanomaterials are shown in Fig. 4(a) and Fig. S2 in the Supplementary Information. The main peaks of Co_0.54_Fe_0.46_OOH were similar to the pure FeOOH, suggesting that there were no phase transformation after Co doping. The elements species of Co_0.54_Fe_0.46_OOH nanostructure were tested by energy-dispersive X-ray spectrum (EDS conducted at three different areas of the sample), confirming Co element was present in the FeOOH nanostructure (Fig. 4(b) and Fig. S3 in the Supplementary Information), and the peaks of Cl element would originate from the Cl species located in the hollandite channels of the FeOOH^27^. The atomic ratios of metals in Co_x_Fe_1−x_OOH (*x* = 0.23, 0.54, 0.77) nanostructures were determined by inductively coupled plasma atomic emission spectroscopy (ICP-AES) measurements (Table S1 in the Supplementary Information).

**Figure 5:**
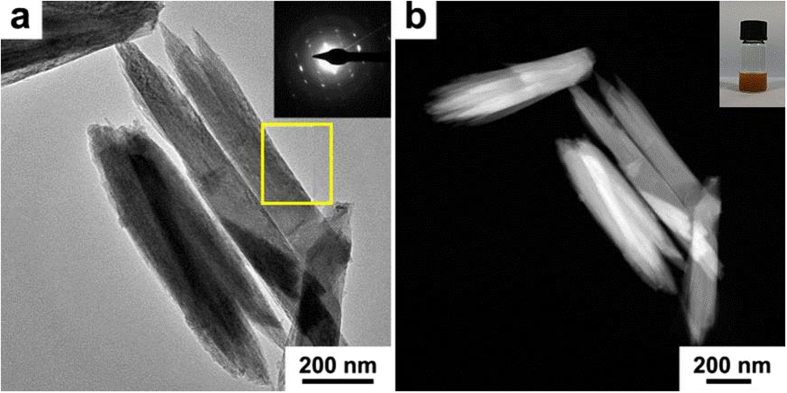
(**a**) TEM and (**b**) HAADF-STEM images of Co_0.54_Fe_0.46_OOH samples. Inset of (**a**) is the SAED pattern of Co_0.54_Fe_0.46_OOH sample (the highlighted yellow area). Inset of (**b**) is the photo of colloidal Co_0.54_Fe_0.46_OOH sample dispersed in absolute ethanol.

TEM and HAADF-STEM characterizations were employed to investigate the morphology and size of Co doped FeOOH nanomaterials (Fig. 5(a,b) and Fig. S4 in the Supplementary Information). As shown in Fig. 5(a) and (b), the rod-like Co_0.54_Fe_0.46_OOH nanostructures were of the average size of ~700 nm ± 100 nm in length and ~100 nm ± 50 nm in width. The SAED pattern (inset of Fig. 5(a)) revealed the Co_0.54_Fe_0.46_OOH nanostructures were of highly crystalline nature. The digital photo in inset of Fig. 5(b) depicts the Co_0.54_Fe_0.46_OOH nanostructures were readily dispersed and highly stable in absolute ethanol. The morphology difference between Co_x_Fe_1−x_OOH and pure FeOOH nanostructures may be due to the presence of Co which could regulate the morphology of FeOOH nanostructures^28^.

### OER electrocatalytic activity of Co_0.54_Fe_0.46_OOH nanostructures

**Figure 6:**
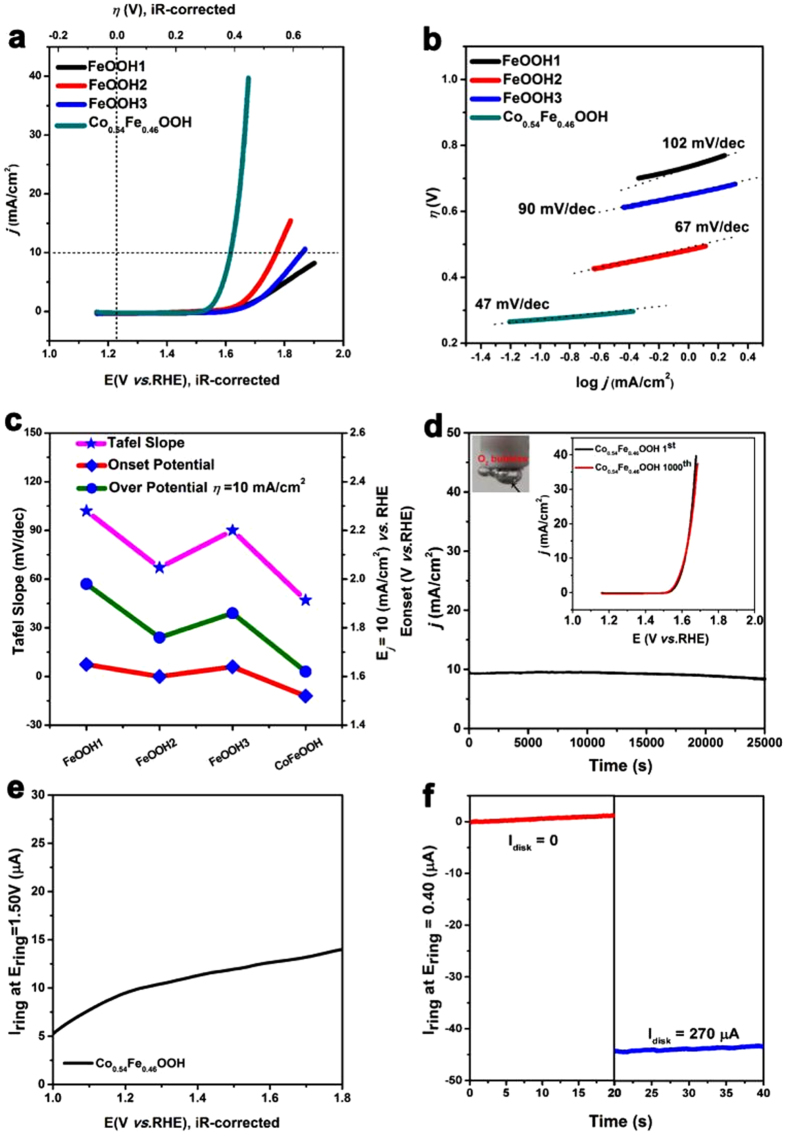
(**a**) Linear Sweep Voltammetry (LSV) of FeOOH1, FeOOH2, FeOOH3, Co_0.54_Fe_0.66_OOH electrode catalysts (The dotteikd lines as reference lines in the Fig. 6). The measurements were carried out with Rotating Disk Electrode (RDE) (loading ∼0.2 mg/cm^2^) at rotation rate of 1600 rpm in O_2_ saturated 100 mM KOH solution at pH = 13. (**b**) Tafel plots (log *j-η*) of FeOOH1, FeOOH2, FeOOH3, and Co_0.54_Fe_0.46_OOH electrodes. (**c**) Comparison of the Tafel slopes, onset potentials and over potentials required to reach a current density of 10 mA/cm^2^ for FeOOH1, FeOOH2, FeOOH3, and Co_0.54_Fe_0.46_OOH electrodes. (**d**) Chronoamperometric measurement for Co_0.54_Fe_0.46_OOH electrode at a fixed applied potential of 1.62 (*vs*. RHE) for 25000 s. Insets in Fig. 6d: (right) LSV for Co_0.54_Fe_0.46_OOH electrode before and after 1000 CV cycles between 1.16 V and 1.96 V at a scan rate of 250 mV/s; (left) an optical photograph of the Co_0.54_Fe_0.46_OOH on the GC electrode during the scans, indicating the production of many O_2_ bubbles on the electrode surface. (**e**) Ring current of Co_0.54_Fe_0.46_OOH on a Rotating Ring-Disk Electrode (RRDE) at a rotation rate of 1600 rpm in O_2_ saturated 100 mM KOH solution at pH = 13 (ring potential 1.50 V). (**f**) Ring current of Co_0.54_Fe_0.46_OOH on an RRDE at a rotation rate of 1600 rpm in O_2_ saturated 100 mM KOH solution at pH = 13 (ring potential 0.40 V).

To investigate the OER catalytic behavior of FeOOH nanostructures, the electrochemical activity of FeOOH1, FeOOH2, FeOOH3, Co_0.54_Fe_0.46_OOH nanomaterials were evaluated based on the above optimized conditions (rotation rate: 1600 rpm in O_2_ saturated 100 mM KOH solution) for the OER performances. For linear sweep voltammetry (LSV) and cyclic voltammograms (CV) curves of studied materials, the ohmic potential drop (iR)^29^ losses arising from the solution resistance were corrected (Fig. 6(a–f) and Fig. S5(a,b) in the Supplementary Information). LSV curves were shown in Fig. 6(a). In contrast, among the FeOOH nanomaterials, Co_0.54_Fe_0.46_OOH exhibited the excellent OER activity with an onset potential of 1.52 V and a sharp rise of the anodic current at the further positive potential, suggesting it’s highly electrocatalytic activity toward OER.

In addition, we also learned about the catalyst properties for different ratio of Co doped nanomaterials under the same conditions (rotation rate: 1600 rpm in O_2_ saturated 100 mM KOH solution), where different ratio of Co doped nanomaterials showed different catalytic activities, among them, Co_0.54_Fe_0.46_OOH had the highest OER catalytic activities (Fig. S5(b) in the Supplementary Information). Importantly, the overpotential of Co_0.54_Fe_0.46_OOH was 390 mV (at a current density of 10 mA/cm^2^), which was lower than those of FeOOH1 (750 mV), FeOOH2 (530 mV), FeOOH3 (630 mV) electrodes. Notably, in comparison with the behavior of most metal oxy-hydroxide nanomaterials in alkaline electrolytes^30^,^31^,^32^,^33^, the overpotential was promising (listed in Supplementary Table S2).

Furthermore, the OER kinetics of the above catalysts was probed by corresponding Tafel plots (log *j* - *η*); where more favorable kinetics and superior catalytic activity were noticeable from the much lower Tafel slopes^34^ The Tafel plots in Fig. 6(b) suggested the kinetics of the electrochemical oxygen evolution on Co_0.54_Fe_0.46_OOH electrode was much faster than others. As shown in Fig. 6(b), the resulting Tafel slopes were found to be ~47, ~67, ~90 and ~102 mV/dec, for Co_0.54_Fe_0.46_OOH, FeOOH2, FeOOH3 and FeOOH1, respectively (Fig. 6(b,c) and Table 1). It should be noted that the Tafel slope of Co_0.54_Fe_0.46_OOH was much smaller than those of other FeOOH1, FeOOH2, FeOOH3, electrodes.

The catalyst stability is always an essential aspect for its property evaluation because durability is crucial for long term utilization^35^. Durability studies of Co_0.54_Fe_0.46_OOH nanomaterials with chronoampero metric measurements were conducted (Fig. 6(d)). After continuous CV scanning, a negligible difference was found between the curves measured at the initial cycle and after 1000 CV cycles (the right inset of Fig. 6(d)). When the potential was fixed at 1.62 V (*vs*. RHE), the catalytic activities remained stable for 25000 s, remaining at 89% of the maximum value. Meanwhile, there were no obvious changes of Co_0.54_Fe_0.46_OOH from the TEM and XPS results (Fig. S5(c) and (d) in the Supplementary Information) after the stability test, which proved the Co_0.54_Fe_0.46_OOH nanomaterials were stable for OER behavior.

To further evaluate OER catalytic activities of electrodes, the mass activity and turnover frequency (TOF) of the above electrodes at *η* = 390 mV (*η* that needed to afford a current density of 10 mA/cm^2^ for Co_0.54_Fe_0.46_OOH electrode) were also presented (Table 1). The calculated mass activity for Co_0.54_Fe_0.46_OOH is 200 A/g, outperforming the other studied catalysts. The constructed Co_0.54_Fe_0.46_OOH electrode exhibited the highest TOF of 0.0225 s^−1^, implying that the metal atom on the crystal surface was catalytically active^36^.

To investigate the reaction mechanism, the rotating ring-disk electrode (RRDE) technique was employed with a Pt ring potential of 1.50 V to oxidize the peroxide intermediates formed on the Co_0.54_Fe_0.46_OOH surface during OER. As shown in Fig. 6(e), a very low ring current (μA scale) was detected, which was three orders of magnitude lower than that of the disk current (mA scale), suggesting a negligible hydrogen peroxide formation and therefore a desirable four-electron pathway for water oxidation: 4OH^−^ → O_2_ + 2H_2_O + 4e^−^ 37. Furthermore, to confirm that the observed current originated from water oxidation rather than other side reactions and to calculate the Faradaic efficiency, an RRDE with a ring potential of 0.40 V was applied to reduce the generated O_2_, rendering a continuous OER (disk electrode) → ORR (ring electrode) process (Fig. S6 in the Supplementary Information)^37^,^38^. With the disk current held constant at 200 μA, O_2_ molecules generated from the Co_0.54_Fe_0.46_OOH catalyst on the disk electrode swept across the surrounding Pt ring electrode that was held at an ORR potential and rapidly reduced. Consequently, a ring current of ~44.70 μA (collection efficiency 0.20) was detected (Fig. 6(f)), which could verify that the observed oxidation current catalyzed by Co_0.54_Fe_0.46_OOH can be fully attributed to OER with a high Faradaic efficiency of 97.30%^39^.

Two possible reasons were responsible for the excellent OER electrocatalytic performances of Co_0.54_Fe_0.46_OOH nanomaterials. The first one was the branch structure on the surface providing many active edge sites, enhanced mass/charge transport capability, easy release of oxygen gas bubbles, and strong structural stability, which are advantageous for OER^40^. The main reason was that the Co-doping in FeOOH nanostructures constituted a desirable four-electron pathway for reversible oxygen evolution and reduction, which is potentially useful for rechargeable metal−air batteries, regenerative fuel cells, and other important clean energy devices^41^. The charge transfer efficiency at the electrode interface was greatly improved after the Co doping into FeOOH nanostructure, which can be demonstrated by Nyquist plots for both catalysts (Fig. S7 in the Supplementary Information).

### Extend the gelatin assisted method to synthesis other materials

Notably, we extend the gelatin assisted soft template method to fabricate lots of other metal oxides such as CoCO_3_ and Ni_3_(CO_3_)(OH)_4_ · 4H_2_O. TEM and HAADF-STEM images of the CoCO_3_ and Ni_3_(CO_3_)(OH)_4_ · 4H_2_O nanostructures are shown in Fig. S8 in the Supplementary Information. Fig. S8(a) and (b) in the Supplementary Information illustrate the belt-like CoCO_3_ nanostructures with the average size of ~700 nm ± 100 nm in length and ~100 nm ± 50 nm in width. Analogously, TEM and HAADF-STEM images of flake-like Ni_3_(CO_3_)(OH)_4_ · 4H_2_O nanostructures with the average size of ~200 nm ± 50 nm in length and ~50 nm ± 10 nm in width are shown in Fig. S8(c) and (d) in the Supplementary Information. The XRD patterns of CoCO_3_ nanostructures in Fig. S9(a) in the Supplementary Information is attributed to a rhombohedra phase of spherocobaltite (JCPDS: 11-0692, space group: R-3C (167) with lattice constants: *a* = 4.65 Å, *b* = 4.65 Å, *c* = 14.95 Å, V = 281.16 Å^3^. Figure S9(b) in the Supplementary Information shows the Co 2p XPS signals of CoCO_3_ nanostructures, and two discernible peaks at 780.90 eV and 796.40 eV were assigned to Co 2p_3/2_ and Co 2p_1/2_, respectively, demonstrating the presence of divalent Co in CoCO_3_^42^. Meanwhile, the XRD patterns of Ni_3_(CO_3_)(OH)_4_ · 4H_2_O nanostructures (Fig. S9(c) in the Supplementary Information) could be ascribed to an orthorhombic phase of nickel oxide hydroxide (JCPDS: 16-0164). Furthermore, Fig. S9(d) in the Supplementary Information depicts the Ni 2p XPS signals of Ni_3_(CO_3_)(OH)_4_ · 4H_2_O nanostructures, and two peaks at 854.90 eV and 872.90 eV were attributed to the Ni 2p_3/2_ and Ni 2p_1/2_, respectively, suggesting the presence of divalent Ni in Ni_3_(CO_3_)(OH)_4_ · 4H_2_O^42^.

## Discussion

To conclude, high-quality FeOOH and Co-doped FeOOH have been synthesized through a gelatin-assisted soft-template wet chemistry process. Subsequently, Co_0.54_Fe_0.46_OOH exhibited higher OER activities, more favorable kinetics, and stronger durability in comparison to those of FeOOH nanostructures. The OER performance was the best among all of the previously reported FeOOH or metal doped FeOOH electrodes and was better than nonmetal OER catalysts, which can be attributed to the branch structure of the Co_0.54_Fe_0.46_OOH nanomaterial on the surface providing many active edge sites, enhanced mass/charge transport capability, easy release of oxygen gas bubbles, and strong structural stability. Meanwhile, a desirable four-electron pathway for reversible oxygen evolution and reduction was generated, which is potentially useful for rechargeable metal−air batteries, regenerative fuel cells, and other important clean energy devices. These Co-doped FeOOH nanostructures should serve as a promising noble-metal-free catalyst for efficient OER in alkaline media.

## Methods

### Chemicals and materials

Ferric chloride hydrates (FeCl_3_ · 6H_2_O, 99.50%, Tianjin Zhiyuan Chemical Company), Nickel chloride hydrates (NiCl_2_ · 6H_2_O, 99.50%, Tianjin Fetching Chemical Company), Cobalt chloride hydrates (CoCl_2_ · 6H_2_O, 99.50%, Tianjin Zhiyuan Chemical Company), Gelatin (C_102_H_151_N_31_O_39_, G7041-500G, 99.00%, Sigma-Aldrich), Urea (CO(NH_2_)_2_, 99.90%, Sigma-Aldrich), and Absolute ethanol (C_2_H_6_O, >99.70%, Guangdong Guanghua Scientific and Technical Corporation) were used as received without further purification.

### Synthesis of FeOOH Nanostructures

In a typical procedure, 27.02 g (100 mmol) of FeCl_3_ · 6H_2_O, 1.00 g of gelatin and 6.06 g (100 mmol) of urea were added into a 100 mL teflon-lined stainless steel autoclave. After stirred for 1 h, the suspension solution in teflon-lined stainless steel autoclave was heated in an electric oven at 80 °C for 21 h. The autoclave was then cooled down to room temperature. The yellow precipitate was collected by centrifugation at 8000 rpm/min for 5 min, washed thoroughly with absolute ethanol, and dried at 65 °C overnight in a vacuum oven. FeOOH2 nanostructures synthetic procedure was similar to FeOOH1 except that urea was not included in the reaction system. FeOOH3 nanostructures synthetic procedure was similar to FeOOH1 except that the concentration of urea was increased to 300 mmol and the reaction time was extended to 46 h.

### Synthesis of Co_x_Fe_1−x_OOH Nanostructures

The synthetic procedure of Co_x_Fe_1−x_OOH was similar to that of FeOOH nanostructures, except that CoCl_2 _· 6H_2_O and FeCl_3 _· 6H_2_O were used with different ratios as the precursors. Different materials, such as: Co_0.23_Fe_0.77_OOH, Co_0.54_Fe_0.46_OOH, Co_0.77_Fe_0.23_OOH were obtained, where Co_0.54_Fe_0.46_OOH exhibited the best OER activity.

### Synthesis of CoCO_3_ Nanostructures

The synthetic procedure of CoCO_3_ was similar to that of FeOOH nanostructures except that CoCl_2_ · 6H_2_O was used as the precursor.

### Synthesis of Ni_3_CO_3_(OH)_4_ · 4H_2_O Nanostructures

The synthetic procedure of Ni_3_CO_3_(OH)_4_ · 4H_2_O was similar to that of FeOOH nanostructures except that NiCl_2_ · 6H_2_O was used as the precursor.

### Characterization

Powder X-ray diffraction (PXRD) patterns of the products were recorded on Rigaku D/MAX-RB (Japan) at a scanning rate of 5°/min from 10° to 70°, using Cu Ka radiation (λ = 1.5406 Å). Transmission electron microscope (TEM) analysis was performed with a Hitachi HT-7700 (Japan) transmission electron microscope operating at 100 kV. Selected area electron diffraction (SAED), high-angle annular dark field-scanning transmission electron microscopy (HAADF-STEM) and energy-dispersive x-ray spectrum (EDS) characterizations were performed with a FeiTecnat G2 F20S-Twin (USA) operated at 200 kV. X-ray photoelectron spectroscopy (XPS) was obtained using an Escalab 250 xi photoelectron spectrometer using Al K radiation (15 kV, 225 W, base pressure ≈5 × 10^−10^ Torr). The amount of atomic ratios of metals in the nanocrystals was determined on Inductively Coupled Plasma Atomic Emission Spectroscopy (ICP-AES) measurements.

### Electrocatalytic Research

Electrochemical measurements were carried out at ambient temperature using a rotating disk electrode (RDE) made of glassy carbon (GC; RDE-3A, 3 mm diameter, 0.07 cm^2^) connected to CHI 760e Electrochemical Workstation (CHI Instruments, Shanghai Chenhua Instrument Corporation, China) in a conventional three-electrode system. The working electrodes were prepared according to the following methods. Typically, 3 mg of catalyst powder and 30 μL Nafion solution (5 wt.%, Sigma-Aldrich) were dispersed in 1.47 mL of DMF solution. Then the mixture solution was sonicated for 2 h to form a homogeneous ink. After that, 7 μL of the dispersion (containing 20 μg of catalyst) was loaded onto the RDE (loading ca. ~0.20 mg/cm^2^). Electrocatalytic performances were conducted in 100 mM KOH (purged with O_2_ for 0.50 h to ensure saturation of the electrolyte) using electrochemical cell setup, with saturated Ag/AgCl electrode (in saturated KCl solution) as the reference electrode and Pt net as the auxiliary electrode, and RDE as the working electrode with a rotation rate of 1600 rpm in O_2_ saturated 100 mM KOH solution (pH = 13). The potentials were reported versus the Ag/AgCl reference electrode, referenced to the RHE through RHE calibration^36^ E_RHE_ = E_Ag/AgCl_ + 0.196 V + 0.0591 pH. Overpotentials (*η*) were calculated based on the formula *η* = E_Ag/AgCl_ + 0.196 V + 0.0591 pH - 1.23 V.

### Calculation of Mass Activity and TOF from Gao and Co-workers

The mass activity (A/g) values were calculated from the catalyst loading m (0.20 mg/cm^2^) and the measured current density *j* (mA/cm^2^) at *η* = 390 mV


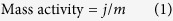


The TOF values were calculated by assuming that every metal atom is involved in the catalysis (lower TOF limits were calculated):


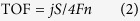


where *j* (mA/cm^2^) is the measured current density at *η* = 390 mV, S (0.07 cm^2^) is the surface area of the GC disk, the constant 4 means 4 electrons/mol of O_2_, F is Faraday’s constant (96485.30 C/mol), and n is the mole of coated metal atom on the electrode calculated from m, the molecular weight of the coated catalysts^37^.

## Additional Information

**How to cite this article:** Zhang, X. *et al*. Effective Construction of High-quality Iron Oxy-hydroxides and Co-doped Iron Oxy-hydroxides Nanostructures: Towards the Promising Oxygen Evolution Reaction Application. *Sci. Rep.* **7**, 43590; doi: 10.1038/srep43590 (2017).

**Publisher's note:** Springer Nature remains neutral with regard to jurisdictional claims in published maps and institutional affiliations.

## Supplementary Material

Supplementary Information

## Figures and Tables

**Table 1:** OER activity data for different catalysts.

Catalysts	Onset Potential [V vs.RHE]	η at J = 10 mA/cm^2^ [mV]	Mass Activity At η = 390mV [A/g]	Tafel Slope [mV/dec]	TOF^a^ at η = 390 mV [s^−1^]
FeOOH1	1.65	750	6.72	102	0.0008
FeOOH2	1.60	530	12.44	67	0.0014
FeOOH3	1.64	630	3.54	90	0.0004
Co_0.54_Fe_0.46_OOH	1.52	390	200.00	47	0.0225

^a^The TOF values were obtained by assuming that every metal atom is involved in catalysis (see the Experiment Section for the calculated method).
