# Supplementary material for: Effective Construction of High-quality Iron Oxy-hydroxides and Co-doped Iron Oxy-hydroxides Nanostructures: Towards the Promising Oxygen Evolution Reaction Application
Source: Sci Rep. 2017 Mar 8;7:43590. doi: 10.1038/srep43590 (PMC5341094; doi:10.1038/srep43590)
Supplement: Supplementary Information [file srep43590-s1.pdf]

Supplementary Information

Effective Construction of High-quality Iron Oxy-hydroxides and Co-doped Iron Oxy-hydroxides Nanostructures: Towards the Promising Oxygen Evolution Reaction Application

Xinyu Zhang[1], Li An[2], Jie Yin[2], Pinxian Xi[2,*], Zhiping Zheng[1,3], and Yaping Du[1,*]

[1] Frontier Institute of Science and Technology jointly with College of Science, State Key Laboratory for Mechanical Behavior of Materials, Xi'an Jiaotong University, Xi'an 710049, P. R. China.

[2] Key Laboratory of Nonferrous Metal Chemistry and Resources Utilization of Gansu Province, State Key Laboratory of Applied Organic Chemistry and College of Chemistry and Chemical Engineering, Research Center of Biomedical Nanotechnology, Lanzhou University, Lanzhou, 730000, P. R. China.

[3] Department of Chemistry and Biochemistry, The University of Arizona, Tucson, AZ 85721-0041, USA.

Correspondence and requests for materials should be addressed to Y. D. (email: ypdu2013@mail.xjtu.edu.cn) or P. X. (email: xipx@lzu.edu.cn)

1. XPS analysis of FeOOH nanomaterials

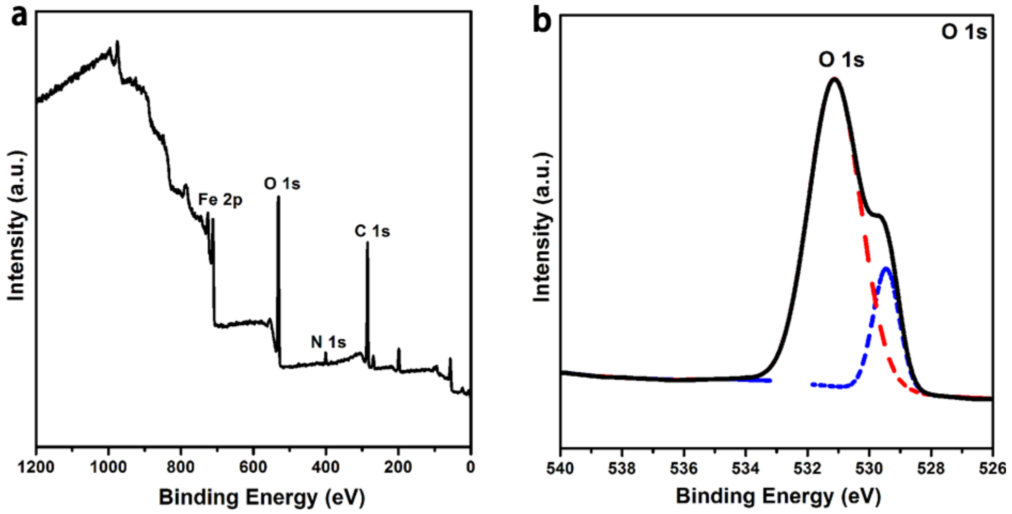

Supplementary Figure S1. (a) XPS survey of FeOOH nanostructures, peaks assignable to core levels of Fe 2p, O 1s, N 1s are identified; (b) XPS pattern of O 1s, the two intense peaks located at 530.0 and 531.6 eV are attributed to the core levels of crystal lattice oxygen and chemisorbed oxygen, respectively[S1].

2. XRD patterns of FeOOH2, $Co_{0.23}Fe_{0.77}OOH$, $Co_{0.54}Fe_{0.46}OOH$, $Co_{0.77}Fe_{0.23}OOH$ nanomaterials.

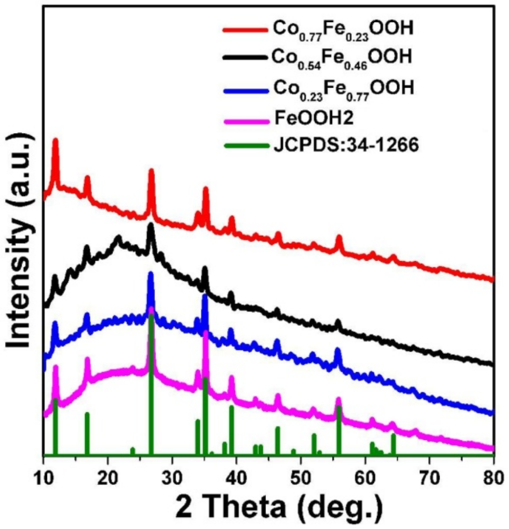

Supplementary Figure S2. XRD patterns of as-prepared FeOOH2, $Co_{0.23}Fe_{0.77}OOH$, $Co_{0.54}Fe_{0.46}OOH$, $Co_{0.77}Fe_{0.23}OOH$ nanostructures.

3. EDS analysis of FeOOH nanomaterials

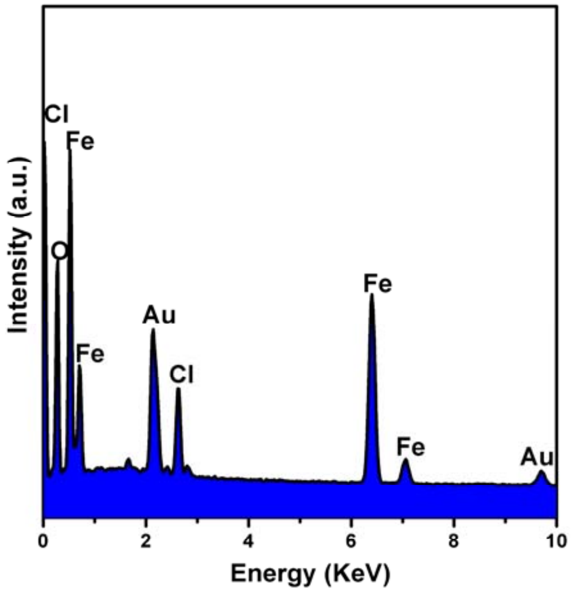

Supplementary Figure S3. The energy-dispersive x-ray spectrum (EDS) spectra of FeOOH. The peaks of Cl element suggested the presence of Cl atoms, which would be located in the hollandite channels of the FeOOH. The peaks of Au element came from sputtering on the surface of sample during the pretreatment.

4. TEM images of FeOOH2, $Co_{0.23}Fe_{0.77}OOH$, $Co_{0.54}Fe_{0.46}OOH$, $Co_{0.77}Fe_{0.23}OOH$ nanomaterials.

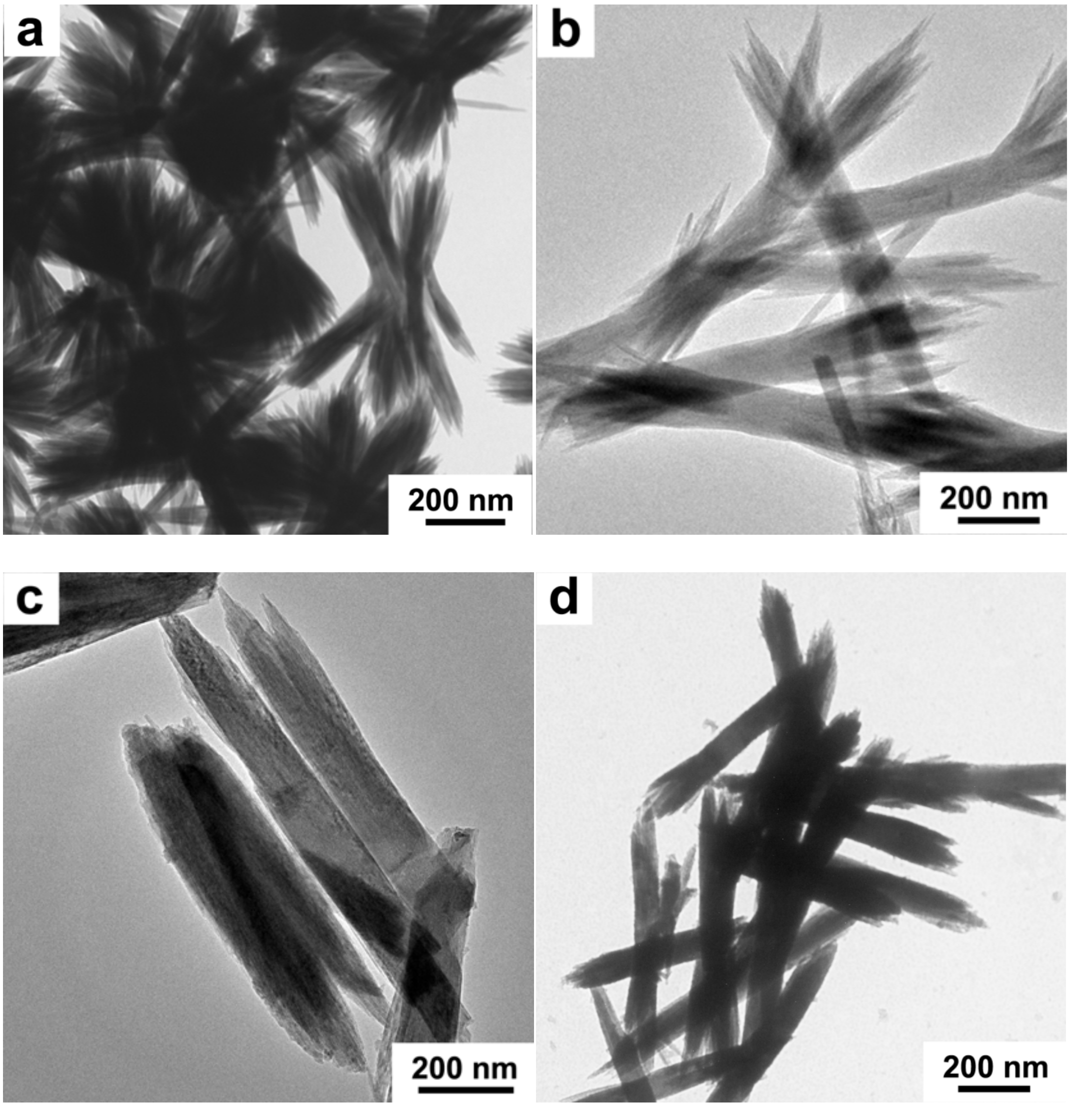

Supplementary Figure S4. TEM images of (a) FeOOH2, (b) $Co_{0.23}Fe_{0.77}OOH$, (c) $Co_{0.54}Fe_{0.46}OOH$, (d) $Co_{0.77}Fe_{0.23}OOH$ nanomaterials.

5. OER analysis of $Co_xFe_{1-x}OOH$ nanomaterials

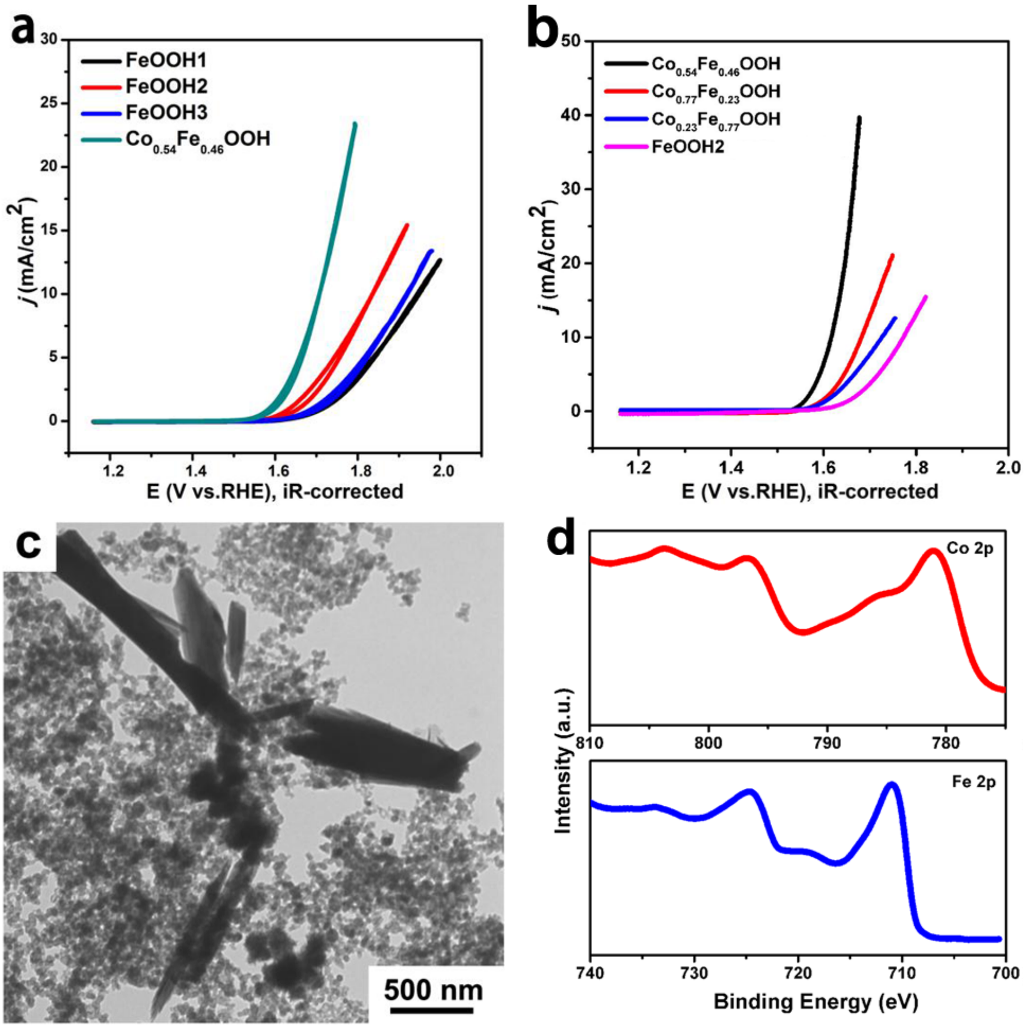

Supplementary Figure S5. (a) iR corrected CV curve of the FeOOH and Co doped FeOOH electrode catalysts; (b) LSV of FeOOH2 and different ratio of Co doped materials: $Co_{0.23}Fe_{0.77}OOH$, $Co_{0.54}Fe_{0.46}OOH$, and $Co_{0.77}Fe_{0.23}OOH$ electrode catalysts; (c) TEM of the $Co_{0.54}Fe_{0.46}OOH$ nanomaterials after the OER process of 25000 seconds; (d) The corresponding XPS spectra of Co 2p and Fe 2p signals after the OER process of 25000 seconds, showing no obvious change of the valence states, which indicates the superior stability of the $Co_{0.54}Fe_{0.46}OOH$ nanostructures.

6. Schematic illustration of OER process

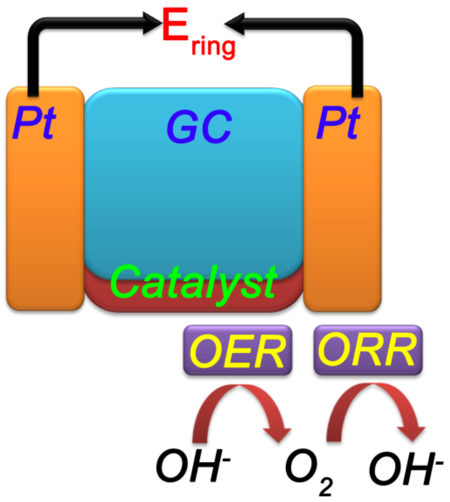

Supplementary Figure S6. Schematic illustration of the continuous with OER (disk electrode) began to ORR (ring electrode) process initiated on a RRDE.

7. Electric resistance of FeOOH and $Co_{0.54}Fe_{0.46}OOH$ nanostructures

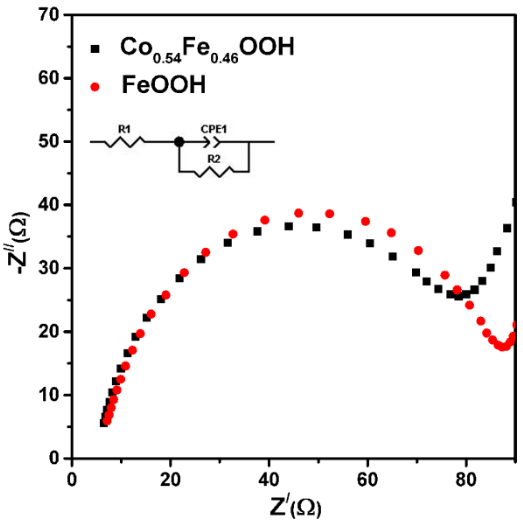

Supplementary Figure S7. Nyquist plots of the FeOOH and $Co_{0.54}Fe_{0.46}OOH$ nanostructures, showing the imaginary part versus the real part of impedance. The charge transfer resistance of $Co_{0.54}Fe_{0.46}OOH$ is about 78 Ω, which is smaller than that of FeOOH nanosheets (88 Ω).

8. Morphologies of $CoCO_3$ and $Ni_3(CO_3)(OH)_4 \cdot 4H_2O$

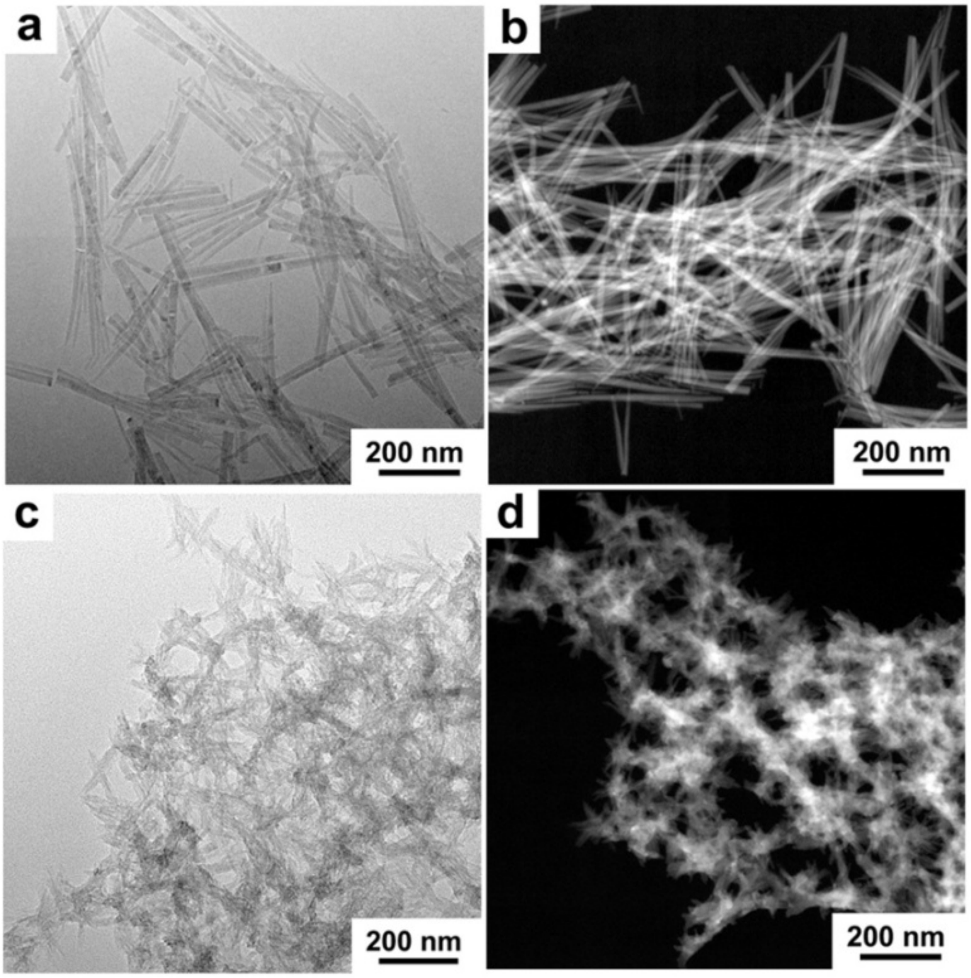

Supplementary Figure S8. TEM and HAADF-STEM images of a, b) $CoCO_3$, c, d) $Ni_3(CO_3)(OH)_4 \cdot 4H_2O$ nanostructures.

9. XRD analysis of $CoCO_3$ and $Ni_3(CO_3)(OH)_4{\cdot}4H_2O$

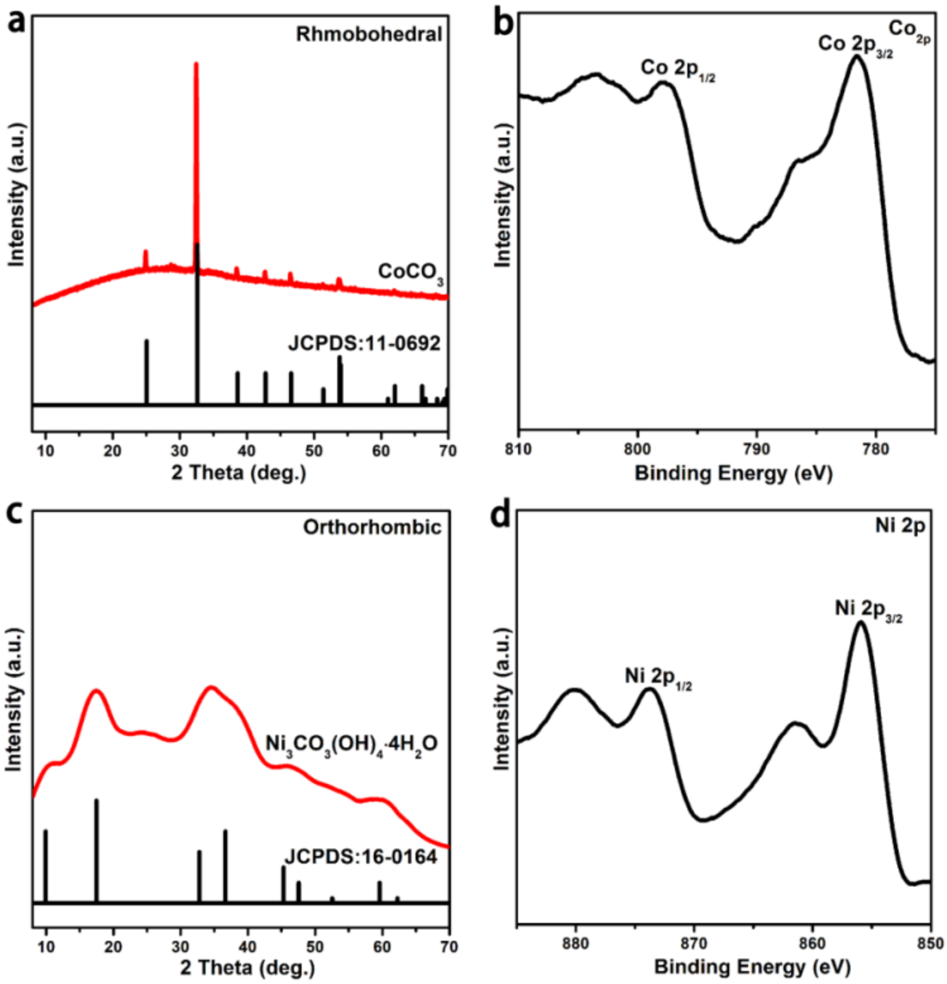

Supplementary Figure S9. (a) XRD patterns of as-prepared $CoCO_3$ nanostructures; (b) XPS patterns of as-prepared $CoCO_3$ nanostructures (Co 2p); (c) XRD patterns of as-prepared $Ni_3(CO_3)(OH)_4{\cdot}4H_2O$ nanostructures; (d) XPS patterns of as-prepared $Ni_3(CO_3)(OH)_4{\cdot}4H_2O$ nanostructures (Ni 2p).

9. ICP analysis of $Co_xFe_{1-x}OOH$ nanomaterials

Supplementary Table S1. ICP results of $Co_xFe_{1-x}OOH$

Catalysts	Co	Fe
**$Co_{0.54}Fe_{0.46}OOH$**	54.53 %	46.47 %
**$Co_{0.23}Fe_{0.77}OOH$**	23.33 %	77.67 %
**$Co_{0.77}Fe_{0.23}OOH$**	77.49 %	23.51 %

10. Comparison OER performance of $Co_xFe_{1-x}OOH$ nanomaterials to other Metal Oxy-hydroxides nanomaterials

Supplementary Table S2. Comparison of OER performance of $Co_{0.54}Fe_{0.46}OOH$ with other electrocatalysts in alkaline electrolytes

Catalysts	Onset Potential [V *vs.* RHE]	η at J=10 mA/cm^2 [mV]	Mass Activity at η=390 mV [A/g]	Tafel Slope [mV/dec]	TOF[a] at η=390 mV [s^{-1}]	Ref.
**MnOOH**	1.72	1000	-	-	-	[S2]
**$Fe_xNi_{1-x}OOH$** (*x*= 0.34, 0.24, 0.1, 0.75, 1)	-	*x*=0 (400) *x*=1 (533) *x*=0.34 (310)	-	60	-	[S3]
**CoOOH**	1.47	330	-	67	-	[S4]
**FeOOH**	-	-	-	47	-	[S5]
**CeO_2/FeOOH**	1.44			92.3		[S6]
**FeOOH/CoOOH/FeOOH**	1.45			32		[S7]
**$Co_{0.54}Fe_{0.46}OOH$**	1.52	390	200	47	0.02	This Work

[a]the TOF values were obtained by assuming that every metal atom is involved in catalysis (see the Experiment Section for the calculated method)

References

S1 Du, Y. P., Zhang, Y. W., Sun, L. D. & Yan, C. H. Efficient energy transfer in monodisperse Eu-doped ZnO nanocrystals synthesized from metal acetylacetonates in high-boiling solvents. *J. Phys. Chem. C.* **112**, 12234-12241 (2008).

S2 El-Deab, M. S., Awad, M. I., Mohammad, A. M. & Ohsaka, T. Enhanced water electrolysis: electrocatalytic generation of oxygen gas at manganese oxide nanorods modified electrodes. *Electrochem. Commun.* **9**, 2082-2087 (2007).

S3 John, R. S., Klaus, S., Trotochaud, L., Bell, A. T. & Tilley, T. D. Electrochemical study of the energetics of the oxygen evolution reaction at nickel iron (Oxy)hydroxide catalysts. *J. Phys. Chem. C.* **119**, 19022-19029 (2015).

S4 Huang, J. H., Chen, J. T., Yao, T., He, J. F., Jiang, S., Sun, Z. H., Liu, Q. H., Cheng, W. R., Hu, F. C., Jiang, Y., Pan, Z. Y. & Wei, S. Q. CoOOH nanosheets with high mass activity for water oxidation. *Angew. Chem. Int. Ed.* **127**, 8846-8851 (2015).

S5 Lu, B. G., Wang, C. Q., Chen, S. L., Yin, J. L., Wang, G. L. & Cao, D. X. A novel composite electrode for oxygen evolution reaction. *J. Solid. State. Electro.* **17**, 2277-2282 (2013).

S6 Feng, J. X., Ye, S. H., Xu, H., Tong, Y. X. & Li, G. R. Design and synthesis of FeOOH/CeO_2 heterolayered nanotube electrocatalysts for the oxygen evolution reaction. *Adv. Mater*. **28**, 4698-4703 (2016).

S7 Feng, J. X., Xu, H., Dong, Y. T., Ye, S. H., Tong, Y. X. & Li, G. R. FeOOH/Co/FeOOH hybrid nanotube arrays as high-performance electrocatalysts for the oxygen evolution reaction. *Angew. Chem. Int. Ed.* **55**, 3694-3698 (2016).
